# An Atypical Bilateral Presentation of Fibrous Dysplasia (FD) in the Mandible: Clinical, imaging and therapeutic characterization

**DOI:** 10.1016/j.ijscr.2021.106049

**Published:** 2021-05-29

**Authors:** Wilber E. Bernaola-Paredes, Henrique Rocha Mazorchi Veronese, Miriã de Andrade Celestino, Ivan Solani Martins, Arthur Ferrari de Arruda, Kleber A. Vallejo-Rosero

**Affiliations:** a Department of Radiation Oncology, A. C. Camargo Cancer Center, Sao Paulo, Brazil; b Department of Stomatology, School of Dentistry, University Center "UNIFAMINAS", Muriaé, Minas Gerais, Brazil; c Department of Oral and Maxillofacial Surgery, Hospital Sirio Libanes, Sao Paulo, Brazil; d Department of Anatomic Pathology, A.C. Camargo Cancer Center, Sao Paulo, Brazil; e Department of Oral and Maxillofacial Surgery, School of Dentistry, Central University of Ecuador, Quito, Ecuador

**Keywords:** Bone diseases developmental, Fibrous Dysplasia of bone, Craniofacial fibrous dysplasia, Oral surgical procedure, Reconstructive surgical procedures, Heterografts

## Abstract

**Introduction and importance:**

Fibrous Dysplasia (FD) is a benign fibro-osseous lesion, characterized by replacement with fibrous connective tissue instead of normal bone. The best treatment option for the condition has not yet been established, although several therapeutic approaches have been reported. The present case report describes the clinical, imaging and therapeutic aspects of an atypical bilateral presentation of FD in the mandible.

**Case presentation:**

A 26-year-old afro-descendent woman, who had previously undergone surgery to remove FD in the right hemimandible, complained of asymptomatic swelling in the left hemimandible. Imaging analysis showed an ipsilateral extensive multilocular mandibular lesion, with thinning of the cortical bone. After diagnosis of FD, complete surgical removal was performed, associated with immediate local reconstruction with xenograft and covering membrane, with primary wound closure.

**Clinical discussion:**

Bilateral presentation of FD is uncommon, and its diagnosis by means of clinical data, imaging and histopathological analysis, is relevant in order to establish the correct therapy.

**Conclusion:**

Complete surgical removal associated with immediate local bone reconstruction, has shown satisfactory clinical results, when adequate follow-up is performed.

## Highlights

• There are few reports based on the atypical bilateral presentation of FD.
• The bilateral presentation of FD in young non-syndromic patients is rare.
• There is a lack of consensus regarding the best treatment for FD.
• The surgical approach is still the best choice for avoiding recurrences.
• Bone grafting after the removal of FD lesions has shown clinical satisfactory results.

## 1 INTRODUCTION

Fibro-osseous lesions (FOLs) are based on a wide group of diseases characterized by the replacement of normal bone by fibrous connective tissue that tends to mineralize gradually. Several lesions that have been described, and it is essential to identify clinical, imaging and histopathological features to establish an accurate diagnosis [1].

Fibrous Dysplasia (FD) is one of the most frequent slow growing FOLs, responsible for approximately 2.5% of all bone lesions and 7% of all benign bone tumors. It is more prevalent in young patients within the first three decades of life, predominantly affecting afro-descendent women [2,3,4]. The lesion has stationary periods and when it progresses into adulthood, it would result in functional and aesthetic changes.

Although its etiology still remains uncertain, FD has been associated with functional mutations in the GNAS1 gene, and is also a factor in the McCune Albright Syndrome (SMA) triad, characterized by the presence of FD, coffee-colored skin stains and metabolic disorders [2].

As regards the clinical features of FD, it could appear as asymptomatic swelling or an increase in the local volume, associated with facial asymmetry, painful symptoms, eye disorders and pathological fractures. On imaging analysis, the lesion appears as an expansive, non-destructive mass, with a well circumscribed cortical and appearance of ground glass, which involves only one anatomical (monostotic) or several (polyostotic) bones [5]. The monostotic variant is the most frequent (80%); however, when the craniofacial bones are involved, the monostotic form is present in 27% of cases, 50% in polyostotic form and 90% of cases in patients with SMA [2]. The maxilla is more frequently affected than the mandible, and in the case of patients with SMA, presentation could be bilateral [5].

On clinical and imaging assessment, differential diagnosis should be established for FD, which include mainly odontogenic tumors (myxoma and ameloblastoma), juvenile ossifying fibroma, keratocyst, central lesion of giant cells and low-grade osteoblastic osteosarcoma [2,4]. In histopathological analysis, immature and irregular bone trabeculae are detected, not surrounded by osteoblasts, scattered in fibrous tissue and with varying degrees of cellularity such as having a narrow, circular, usually hook-shaped appearance or similar to that of Chinese letters [6]. However, based on an entire analysis, cases of malignant transformation have been observed [2].

The treatment of FD is generally based on surgical management. However, there is still no consensus on the best approach in order to avoid recurrences. Therefore, adequate surgical removal is the best approach commonly used, because it has been associated with lower rates of recurrence [3]. In fact, surgical techniques such as curettage, peripheral osteotomies, osteoplasty with local reconstruction based on the placement of bone grafts after surgery have been described in the literature [5].

The present case report aimed to describe the clinical, imaging and therapeutic features of bilateral fibrous dysplasia in the mandible in a young patient. This study was reported in line with the SCARE 2020 criteria [7].

## 2 Case report

A 26-year-old afro-descendent woman, with the complaint of asymptomatic swelling in the left hemimandible, which was previously detected in the imaging examination. Without relevant data from her medical history, the patient informed our team that had been submitted to surgical resection for removal of an FD lesion on the opposite side (right hemimandible).

During extraoral examination, slight facial asymmetry was observed on the left side, with local swelling in the submandibular region, without the presence of lymph node enlargement in the region. Moreover, on intra-oral examination, an increase in buccal and lingual bone plate volume was detected, extending from tooth 33 to tooth 37 on the ipsilateral side, without tooth mobility and pain after manual palpation and percussion.

**Fig. 1:**
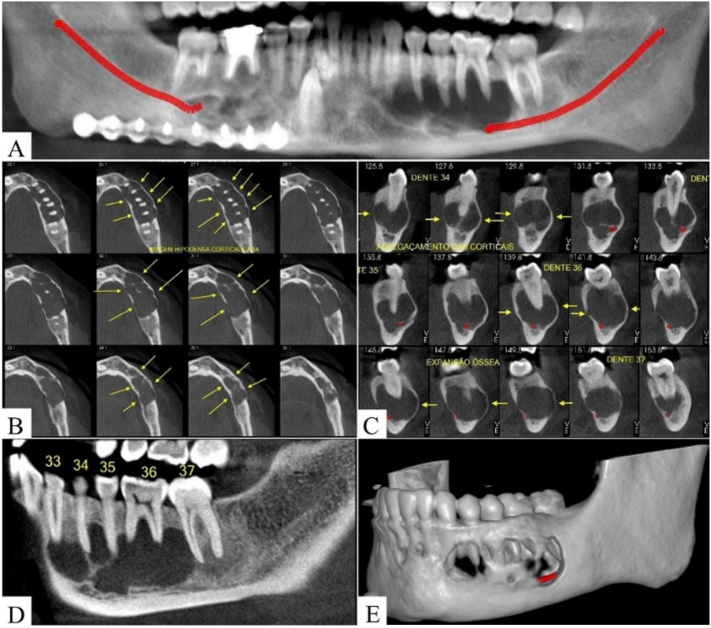
Initial CT imaging analysis. (A) CT in panoramic reconstruction shows single, extensive and mixed lesion in left mandibular region, closer to inferior alveolar nerve (red line). Axial (B) and sagittal (C) sections showed bulging and thinning of buccal and lingual plates (yellow arrows). (D) Lateral view and (E) 3D mandibular reconstruction showed extensive volume of FD.

On orthopantomography (OP) (Fig. 1A) and Cone beam computed tomography (CT) analysis with sagittal (Fig. 1B) and axial (Fig. 1C) slices, a single, extensive, and multilocular mandibular lesion with the appearance of “frosted glass” was observed, closer to the left mandibular basal-edge, which basically promoted the thinning of the lingual plate. After the 3D imaging reconstruction, a destructive lesion with irregular edges and no encapsulated membrane was visualized, without root resorption of teeth (Fig. 1D).

Based on clinical data and imaging features collected, the initial diagnosis of fibro-osseous lesion was established; however, differential diagnosis was considered such as ameloblastoma, keratocystic odontogenic tumor, and central lesion of giant cells.

**Fig. 2:**
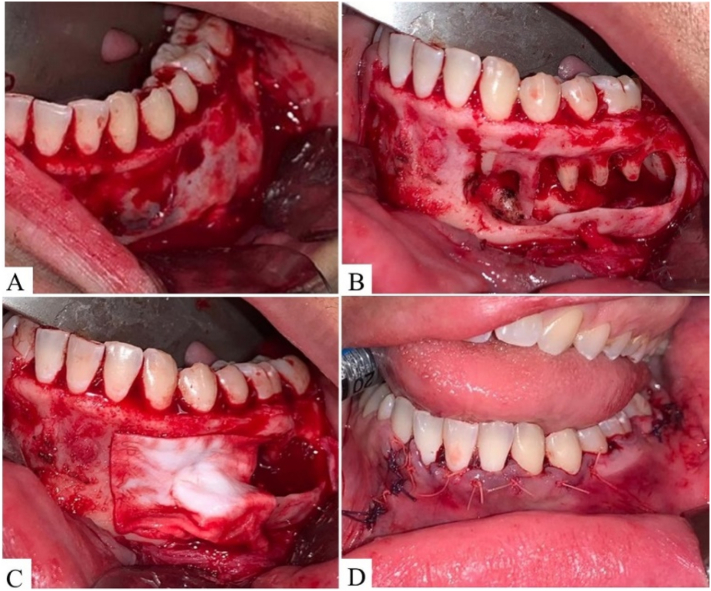
Surgical approach. (A) Exposure of bone lesion by performing modified Newman incision. (B) Complete removal by curettage and peripheral osteotomy of surrounding bone, with preservation of roots of associated teeth. (C) Osteoinductive overlay membrane on xenograft for immediate local reconstruction after removal. (D) Primary wound closure by suture.

**Fig. 3:**
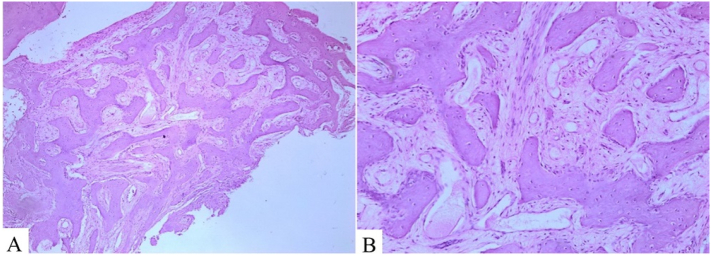
Histopathological analysis. Bone trabeculae without visible osteoblastic rimming within fibrous stroma with bland monotonous spindle cells observed by microscopy at 40× (A) and 100× (B) magnification.

Excisional biopsy that consisted of exposing the lesion by performing a Newmann modified incision (Fig. 2A) for final diagnostic and therapeutic purposes. Thus, complete removal by curettage and peripheral osteotomy of the surrounding bone were performed (Fig. 2B). After diagnostic confirmation of FD, immediate local reconstruction was performed with placement of xenograft (Bio-OSS, Geistlich, Sao Paulo, Brazil) covered with collagenous membrane (Bio-Gide, Geistlich, Sao Paulo, Brazil) (Fig. 2C), followed by primary wound closure (Fig. 2D). In histopathological analysis, the presence of trabecular bone tissue without visible osteoblastic rimming was observed, amid fibrous stroma with monotonous spindle cells without atypical mitosis (Fig. 3A–B) that confirmed the diagnosis of FD.

**Fig. 4:**
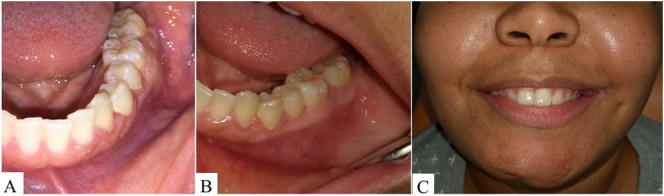
Complete intraoral healing after three (A) and six months of follow-up (B). (C) Absence of facial asymmetry on the left side after six months.

**Fig. 5:**
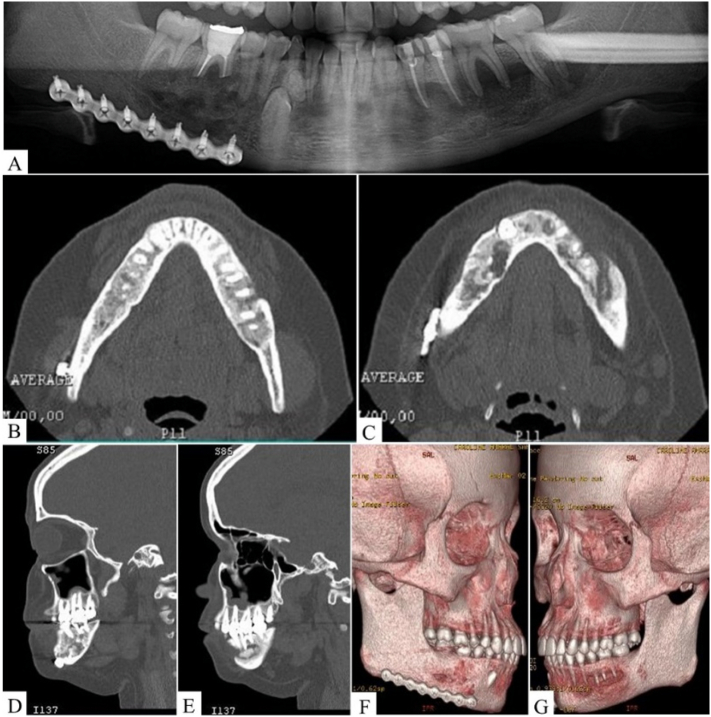
Follow-up imaging analysis. OP performed fifteen days after surgery (A). Axial (B–C) and sagittal (D–E) CT slices after six months showing evidence of bone repair. 3D reconstruction showed bone regeneration of both regions, the right side after two years (F) and left side after six months (G) of follow-up.

After three and six months of follow-up, complete intraoral healing (Fig. 4A–B) and absence of facial asymmetry on the left side (Fig. 4C) were observed, and teeth associated with the initial lesion were submitted to root canal treatment in order to avoid further local recurrences Moreover, axial (Fig. 5A–B) and sagittal (Fig. 5C–D) CT sections showed a mixed area corresponding to intra-osseous healing process and new bone, confirmed by the 3D imaging reconstruction of both hemimandibles (Fig. 5E, F).

## 3 Discussion

FD is a rare benign FLO that can involve a single bone (monostotic) or several (poliostotic) bones, frequently appearing with unilateral distribution. However, bilateral presentation in the same bone is rare, observed in patients with SMA [5,8]. In this case report, a rare FD lesion with bilateral presentation in the mandible was shown in a non-syndromic patient.

Although the nature of DF is benign, this condition can become malignant, however, in rare cases [2,6,9]. Thus, the most important differential diagnosis to consider would be low-grade osteosarcoma, which has been described in the literature as the most frequent transformation of these lesions over time; however, fibrosarcoma and chondrosarcoma malignancies have also been described [2,10]. Factors such as hypercellularity, nuclear atypia, pleomorphism, increased mitotic activity and high percentage of proliferative biomarker Ki67 may suggest malignancy and worse prognosis [6,10]. Indeed, correct diagnosis based on clinical, imaging and histopathological features would enable the establishment of an adequate therapeutic plan and appropriate follow-up for the purpose of detecting possible malignant transformations and avoiding recurrences, and would thus promote a favorable prognosis.

High recurrence rates have been associated with inadequate surgical techniques [2]. In a recent retrospective clinical-pathological study conducted by Özşen et al. [6], recurrences were observed after limited bone curettage, since they were associated with incomplete removal of deregulated tissue, which would lead to preservation of mutated somatic cells, such as the GNAS1 gene that constitutes the main mutational mechanism linked to the condition [6,8,9].

As yet, the treatment modality described for FD that would be best, has not yet been fully established [11]. In asymptomatic cases and without evidence of deformities, clinical and imaging follow-up has been recommended, without further surgical interventions. Whereas in symptomatic presentations and those with bone changes that decrease function, surgical removal is the first choice. After surgery, local reconstruction with the use of bone grafting has shown satisfactory clinical results [3,5,6]. Moreover, recent target therapies based on the use of antiresorptives and monoclonal antibodies have been considered for clinical stabilization, reduction in rates of progression and control of symptoms [3]. In the present case, curettage and peripheral osteotomy of the surrounding bone were performed for complete removal of the lesion, and for remnant bone preservation. Local reconstruction was performed with the use of xenograft bone covered with a membrane to promote bone repair, evidence of which was shown both clinically and by imaging assessment, after a 06-month period of follow-up.

## 4 Conclusion

Bilateral presentation of FD is rare, however, it could be observed in young non-syndromic patients. The diagnosis of FD continues to be a challenge because of its clinical, imaging and histopathological similarities with other fibro-osseous lesions; in advanced cases with long-term evolution, malignant transformation could be considered. Treatment remains controversial, however, complete removal by resective surgery and immediate reconstructive procedures such as bone grafting and covering membranes placed over remnant bone have been shown to achieve satisfactory clinical results after adequate follow-up.

## Ethical approval

All procedures performed in studies involving human participants were in accordance with the ethical standards of the institutional and/or national research committee and with the 1964 Helsinki declaration and its later amendments or comparable ethical standards. This report was exempt for ethical approval because of the use of medical data records of the patient without exposure of her identity or photos that shows her facial identification or characteristics.

## Sources of funding

This work was supported by the National Council for Scientific and Technological Development of Brazil (140071/2019-9). The author WEBP is student fellow and supported with a scholarship provided by the National Council for Scientific and Technological Development of Brazil (CNPq).

## CRediT authorship contribution statement

All authors have contributed in the each step for writing this paper, participating in the whole process to retrieve medical information, review of the literature, and writing of each issue included.

Wilber Edison Bernaola-Paredes and Henrique Rocha Mazorchi Veronese have written and selected the topics for structuring this case report. Moreover, he did and worked in the Introduction and Discussion issues. On the other hand, Kleber A. Vallejo-Rosero, Miriã de Andrade Celestino, Ivan Solani Martins and Arthur de Arruda Ferrari has done the literature review in order to support our discussion and introduction. Wilber E. Bernaola-Paredes and Ivan Solani Martins were the oral and maxillofacial surgeons who performed lesion removal.

## Guarantor

Wilber Edison Bernaola-Paredes

Kleber A. Vallejo-Rosero

## Research registration

Not applicable.

## Consent

Written informed consent was obtained from the patient for publication of this case report and accompanying images. A copy of the written consent is available for review by the Editor-in-Chief of this journal on request.

## Provenance and peer review

Not commissioned, externally peer-reviewed.

## Declaration of competing interest

None declared.
